# Trial watch: an update of clinical advances in photodynamic therapy and its immunoadjuvant properties for cancer treatment

**DOI:** 10.1080/2162402X.2023.2226535

**Published:** 2023-06-18

**Authors:** Mafalda Penetra, Luís G. Arnaut, Lígia C. Gomes-da-Silva

**Affiliations:** CQC - Coimbra Chemistry Center, Universidade de Coimbra, Coimbra, Portugal

**Keywords:** anti-tumor immunity, cancer, clinical trials, photodynamic therapy

## Abstract

Photodynamic therapy (PDT) is a medical treatment used to target solid tumors, where the administration of a photosensitizing agent and light generate reactive oxygen species (ROS), thus resulting in strong oxidative stress that selectively damages the illuminated tissues. Several preclinical studies have demonstrated that PDT can prime the immune system to recognize and attack cancer cells throughout the body. However, there is still limited evidence of PDT-mediated anti-tumor immunity in clinical settings. In the last decade, several clinical trials on PDT for cancer treatment have been initiated, indicating that significant efforts are being made to improve current PDT protocols. However, most of these studies disregarded the immunological dimension of PDT. The immunomodulatory properties of PDT can be combined with standard therapy and/or emerging immunotherapies, such as immune checkpoint blockers (ICBs), to achieve better disease control. Combining PDT with immunotherapy has shown synergistic effects in some preclinical models. However, the value of this combination in patients is still unknown, as the first clinical trials evaluating the combination of PDT with ICBs are just being initiated. Overall, this Trial Watch provides a summary of recent clinical information on the immunomodulatory properties of PDT and ongoing clinical trials using PDT to treat cancer patients. It also discusses the future perspectives of PDT for oncological indications.

## Introduction

In 1900, a German medical student, named Oscar Raab, discovered by accident that acridine orange dye could kill protozoa in the presence of light. Later, in the second half of the twentieth century, Thomas Dougherty furthered this discovery by finding that hematoporphyrin derivatives (HpD) obtained from hemin in blood could be used to treat solid tumors with the aid of light. This led to the inception of modern Photodynamic Therapy (PDT)^1,2^.

PDT is a medical treatment that requires a molecule (named photosensitizer) that is activated by visible or near infrared light. When the photosensitizer is photoactivated in the presence of molecular oxygen (O_2_), reactive oxygen species (ROS) are promptly generated. The acute oxidative stress associated with PDT can be used to selectively kill cancer cells and other abnormal cells in the body^3,4^. In clinical practice, PDT is a two-step treatment modality that begins with the intravenous administration (iv) of the photosensitizer or its topical application to the skin. After a specific period of time, known as drug-to-light interval (DLI), the photosensitizer is activated at tumor sites using an external light source (*e.g*. laser or LED) at a wavelength that matches the lowest energy band of the photosensitizer. Ideally, this wavelength should be between 650 and 850 nm to allow deeper light penetration into the tumor tissues^3^. Clinically approved photosensitizers are typically administered for several hours (or even days) in advance of the light treatment. These protocols with a long DLI allow enough time for the photosensitizer to be internalized by the cancer cells, meaning that generated ROS can directly damage and kill the cancer cells (cellular-PDT). In contrast, PDT with a short DLI (*e.g*. 15 minutes) destroys the tumor vasculature (vascular-PDT), killing cancer cells indirectly by interrupting their supply of nutrients and oxygen^3,5,6^.

PDT is mainly being used to treat skin cancer, such as basal cell carcinoma, and other skin-related disorders, like actinic keratoses and acne rosacea^7^. It is also approved by various regulatory agencies for lung, esophageal, and head and neck cancers (Table 1). Clinical and preclinical evaluation of PDT has shown promising results, which contribute to the growing awareness of PDT as a potential cancer treatment. PDT offers the advantages of being minimally invasive and generally well-tolerated, with temporary light sensitivity being its most common side effect. In fact, the PDT market is expected to experience significant growth in the upcoming years, with a compound annual growth rate estimated by different market research companies to be > 5% in the next few years^8,9^.

**Table 1.** Photosensitizers for PDT of cancer that have been approved for clinical use by regulatory agencies in Europe, America or Japan.

Photosensitizer	Wavelength (nm)	ε (M^−1^ cm^−1^)	Approved clinical application	PDT conditions
Porfimer sodium (Photofrin®)	630	3.0x10^3^	Esophageal cancer, Endobronchial cancer, High-Grade Dysplasia in Barrett’s Esophagus	USA, Canada
Temoporfin/m-THPC (Foscan®)	652	3.0x10^4^	Advanced Head and neck cancer	European Union
Talaporfin/Chlorin e6 (Laserphyrin®)	664	4.0x10^4^	Early stage lung cancer, esophageal cancer esophageal cancer	Japan
5-ALA (Levulan®) and its methyl (Metvix®) or benzyl (Benzvix®) ester derivatives (*)	635	5.0x10^3^	Actinic keratoses (#)	USA
IR700 linked to cetuximab (cetuximab sarotalocan)	690	2.1x10^5^	Head and neck cancer	Japan

(*) These molecules are pro-drugs of the photosensitizing agent, **PpIX**.

**(#)** Actinic Keratoses is a pre-cancerous lesion that can evolve to cancer if left untreated.

The clinical use of PDT started in 1993 with the approval of HpD, marketed as **Photofrin® (porfimer sodium)**, in Canada for the treatment of bladder cancer. This was followed by its approval in Japan (1994) and by the Food and Drug Administration (FDA) for the treatment of esophageal cancer (1995)^1,10^. This was an important milestone in the PDT field. However, **porfimer sodium** has several limitations that compromised its widespread acceptance by the medical community. In fact, **porfimer sodium** is not a pure compound, but instead, it is a complex mixture of HpD dimers and oligomers with poor water-solubility. In clinical use, its photoactivation is carried out at 630 nm, which is associated with low tissue penetration. Its low molar absorption coefficient (ε_630 nm_ 3000 M^−1^ cm^−1^) requires high PDT regimens (PS = 2 to 5 mg/kg, DL = 100 to 200 J/cm^2^) to obtain therapeutic effects. Additionally, its slow body clearance (half-life of 21.5 days) is likely its biggest limitation as it is associated with prolonged photosensitivity that requires more than one month of sunshade^10–13^.

With the intention of surpassing the limitations of **porfimer sodium** toward a better photosensitizer, researchers have attempted to design new molecules that fulfill the properties of an ideal photosensitizer. Some progress has been made which led to the emergence of second-generation photosensitizers such as **temoporfin (Fotolon®)** and **talaporfin (Foscan®)** chlorins which are characterized by high absorptions at 650–660 nm: ε_650 nm_ = 39000 M^−1^ cm^−1^ in EtOH; ε_652 nm_ = 23000 M^−1^ cm^−1^ in H_2_O for **temoporfin** and ε_654 nm_ = 40000 M^−1^ cm^−1^ in PBS for **talaporfin**^14,15^. PDT with **temoporfin** was approved for the treatment of advanced head and neck cancer by the European Medicines Agency (EMA) in 2001 but its market authorization was declined by the FDA. It requires lower PDT regimens (PS = 0.1 to 0.3 mg/kg and LD = 8 to 12 J/cm^2^), which denotes its higher potency when compared to **porfimer sodium**. On the other hand, **talaporfin** is only approved in Japan for the treatment of advanced lung cancer (2004) and esophageal cancer (2015). Patients submitted to PDT with **temoporfin** or **talaporfin** are advised to avoid light exposition for *c.a.* 2 weeks^16,17^.

**Sulfonated aluminum phthalocyanine (AlPcS)** is a water-soluble derivative of aluminum phthalocyanine that has been modified with sulfonate groups (-SO3-) to enhance its solubility. It has a strong absorption peak at 680 nm (ε_672 nm_ ~20 × 10^5^ M^−1^ cm^−1^ in PBS)^18^. It is in clinical use for cancer treatment of different histological origin but only in Russia^19,20^.

Another important milestone in PDT was the approval of the first targeted photosensitizer, which is often defined as a characteristic of “third generation” photosensitizers. This class of PS intends to enhance the selectivity and/or cellular uptake of the photosensitizing agents by means of targeting moieties (*e.g*. monoclonal antibodies) that specifically bind to receptors overexpressed on tumor cells (active targeting)^21,22^. **Cetuximab saratolacan (Akalux®)** is the bioconjugate of the silicon-phthalocyanine derivative **IRDye700DX** (best known as **IR700**) conjugated to cetuximab. The latter is an FDA-approved antibody targeting the epidermal growth factor receptor (EGFR), which is overexpressed in many types of cancer. This bioconjugate was approved in 2019 for the treatment of advanced and recurrent head and neck cancer in Japan^23^. The targeting conjugate has a peak of absorption at 690 nm (ε_689 nm_ = 2.1 × 10^5^ M^−1^cm^−1^)^24^. Tumor illumination is performed 24 h after the iv administration of the targeting conjugate. This DLI is expected to favor the accumulation of the targeting conjugate at the surface of EGFR^+^ cells meaning that upon irradiation, only EGFR expressing cells are selectively destroyed^25^.

Precursors of the endogenous photosensitizer **protoporphyrin IX**, such as **5-aminolevulinic acid (Levulan®)** and its methyl **(Metvix®)**, hexyl **(Hexvix®)** or benzyl **(Benzvix®)** ester derivatives have been used with considerable success namely in skin cancer (basal cell carcinoma) and other skin-related diseases (actinic keratoses)^26,27^. In this case, **5-ALA** and its derivatives are metabolized into **protoporphyrin IX** (the photosensitizing agent) through a series of reactions involving enzymes of the heme biosynthetic pathway. **5-ALA** and its derivatives exhibited reduced skin photosensitivity however, their low absorption at 635 nm (ε_635 nm_ = 5000 M^−1^cm^−1^, in PBS) limits light penetration depth to ~2 mm^11^. For this reason, **5-ALA** and its derivatives are mainly used for skin diseases upon topical administration. In some circumstances, the limitation of the short light penetration can be overcome by administering the **5-ALA** derivative close to the target. This is the case of the instillation of **Hexvix®** in the bladder, which allows for its uptake by cancer cells in the bladder and improved detection of urothelial carcinoma by fluorescence cystoscopy^28^.

Although without clinical indication for cancer treatment, **verteporfin (Visudyne®)** should be mentioned owing to its success for the treatment of age-related macular degeneration (AMD) both in USA and in Europe (2000)^29^. Considering that the target is the ocular vasculature, **verteporfin** activation is conducted immediately 15 min after its administration (vascular-PDT). **Verteporfin** has a peak of absorption at 689 nm (ε_692 nm_ = 13500 M^−1^cm^−1^ in PBS) and an elimination half-life of 5–6 h, which reduces the period of skin photosensitivity to less than 48 h^30,31^. **Verteporfin** has been evaluated for the treatment of cancer (namely non-melanoma skin and pancreas cancer) in numerous clinical trials^32^.

## Preclinical evidence of anti-tumor immunity mediated by PDT

PDT is gaining increasing attention due to its immunomodulatory properties, which can instruct the host immune system to recognize and effectively eliminate cancer cells^3,33^. The increased awareness of PDT as a new form of immunotherapy is based on a large body of preclinical evidence that have been collected in the last two decades. The initial indications of the immunomodulatory properties of PDT came from vaccination experiments either by using lysates from cancer cells submitted to PDT (PDT-based lysates vaccines) or by directly using PDT-killed cancer cells (PDT-based whole cell vaccines)^34,35^. Cancer cells stressed with PDT have also been used to directly activate dendritic cells (DCs) (PDT-based DC vaccines) which induces anti-tumor immune responses robust enough to significantly impair tumor growth^36^. Other preclinical evidences show the involvement of the host immune system in the PDT therapeutic efficacy. For instance, numerous studies using different photosensitizing agents have shown that PDT produces better therapeutic outcomes in immunocompetent mice rather than in immunocompromised counterparts. Most of these studies use Balb/c nude mice which lack T cells. Similar observations have been attained upon depletion of T cells, namely CD8^+^ T cells, by means of specific antibodies^6,37–42^. The reduced efficacy in immunocompromised mice, or upon CD8^+^ T cells depletion, reveals the importance of T cells for the efficacy of PDT and suggests an important contribution of the adaptive immune system. In line with these observations, researchers have observed that tumor-bearing mice that have been cured with PDT acquired immunological memory. The latter is robust enough to confer protection against subsequent rechallenge with live cancer cells^37,38,43–50^. Anti-tumor immunity is of utmost importance due to its capacity to identify and eliminate distant and non-illuminated metastases. This has been demonstrated in several pseudo-metastatic models, including double-tumor models where mice carry two tumors (one in each flank), and primary tumor-bearing mice with lung metastases that result from the intravenous injection of cancer cells. In these cases, tumor regression can be observed at both illuminated and non-illuminated tumor lesions^6,38,45,46,48^.

The reason behind the increased antigenicity and immunoadjuvanticity of PDT-stressed cells is not yet fully understood but appears to be independent of the chemical structure or the intracellular tropism of the photosensitizers. The immunological consequences of PDT are likely related with its ability to induce a type of cell death broadly known as immunogenic cell death (ICD). ICD is considered as any form of cell demise that can mount an adaptive immune response in immunocompetent syngeneic hosts without the need of any immunoadjuvant^51^. Cell-based assays show that PDT (performed with a variety of photosensitizers) induces cell death by different mechanisms (apoptosis, necrosis, autophagy, paraptosis, necroptosis, etc.). Independently of the main form of cell death, PDT-stressed cells appear to have the aptitude to release/expose, in a spatial-temporal manner, a specific set of intracellular molecules that acquired immune-stimulatory effects when located outside of the cells. These molecules, named as damage-associated molecular patterns (DAMPs), are recognized by pattern recognition receptors (PRRs) expressed on immune cells which result in the activation of immune cell of the different arms of immune system. Altogether, DAMPs activate the recruitment of immune cells to the tumor bed, where they facilitate the presentation of tumor associated antigens (TAA) to antigen presenting cells (APC)^33^. In this regard, neutrophils have been recognized to play an important role in the development of PDT-mediated anti-tumor immunity. Infiltration of neutrophils into PDT-treated tumors accompanied with neutrophilia (enhanced number of neutrophils in the peripheral blood) is reported for different photosensitizing agents soon after their photoactivation^42,48,52–58^. Several studies have revealed the importance of neutrophils for the efficacy of PDT. Depletion of neutrophils using specific antibodies or using mice defective in neutrophil homing to peripheral tissues (CXCR2^−/−^ mice) significantly impaired the curative effect of PDT. This effect was correlated with reduced number of activated cytotoxic T cells. In fact, PDT stimulates the expression of MHC class II not only in DCs but also in neutrophils. Antigen uptake by these cells promotes their maturation, facilitating their migration to lymph nodes. In case of neutrophils, their migration to tumor-draining lymph nodes is regulated by Th17 T cells. In the lymph nodes, presentation of tumor antigens prime tumor-specific cytotoxic CD8^+^ T cells^58^. While the importance of CD8^+^T cells for the anti-cancer effect of PDT is strongly supported by several preclinical studies, the precise involvement of CD4^+^ T cells and B cells remains elusive, with some studies reporting contradictory results^39,42,48^. Overall, an extensive number of preclinical studies show that PDT elicits an extensive list of immunological events that engage a diverse array of innate and adaptive immune cells. The role of these cellular components in the promotion of PDT-induced anti-tumor immunity has been extensively reviewed elsewhere^33,59–61^.

**Figure 1.**
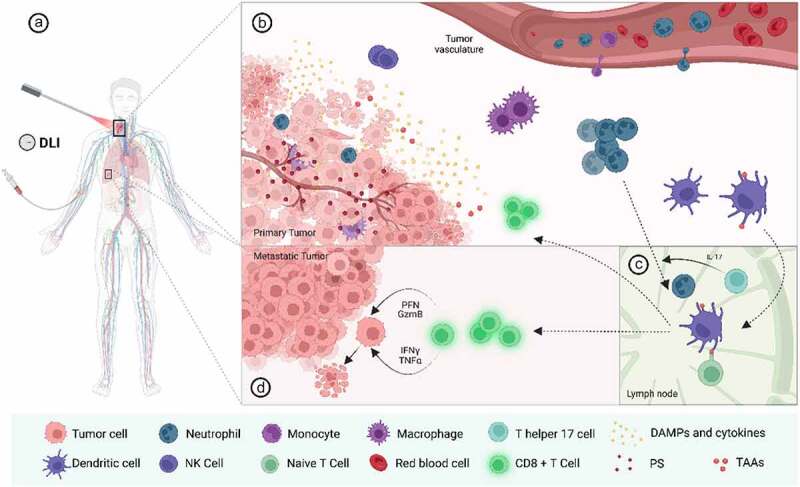
Activation of anti-tumour immunity after PDT based on preclinical and clinical data. (a) Illustrative example of a patient with head and neck cancer with lung metastases submitted to PDT treatment. The photosensitizer is intravenously administered and after a certain time interval, the target tumour is irradiated with the appropriate wavelength to activate the PS. (b) Irradiation of the tumour leads to the activation of the photosensitizer followed by ROS production and cell destruction by various modes of cell death (e.g. apoptosis, necrosis, necroptosis, paraptosis, autophagy, etc.). Some of these forms of cell demise hold immunological features being broadly known as immunogenic cell death (ICD). ICD results in the exposure/secretion of damage-associated molecular patterns (DAMPs) such as, calreticulin (CRT), high mobility group box 1 (HMGB1), adenosine triphosphate (ATP), annexin A1 (ANXA1), and heat-shock proteins (HSP). PDT-stressed cells also release cytokines and chemokines (e.g. IL6), which together with the DAMPs, leads to a strong and acute inflammation and the recruitment of innate immune cells (e.g. neutrophils, monocytes/macrophages and natural killer cells) to the tumour bed. Neutrophils are important for the activation of adaptive immunity after PDT, as some evidence suggests that they can also act as antigen presenting cells after PDT. Neutrophils and DC engulf tumour-associated antigens (TAAs) released by dying tumour cells, transitioning from an immature to a mature state. They then migrate to the lymph nodes, which in the case of neutrophils appears to be regulated by Th17 T cells. (c) In the lymph nodes, mature DCs, and neutrophils, prime naive T cells by presenting antigens peptides on their surface. This leads to activation and cloning expansion of CD8^+^ T cells. (d) Activated CD8^+^ T cells are then released from the lymph nodes and enter in the bloodstream being able to recognise remaining tumour cells, both at the primary (and illuminated) tumour or distant metastases. To kill tumour cells, activated CD8^+^ T cells secrete several cytokines, such as perforin P, granzyme B, INF-γ and TNF-α, which can directly kill tumour cells. Figure created with BioRender.com.

## Clinical evidence of antitumour immunity mediated by PDT

While preclinical studies have extensively demonstrated the anti-tumor immunity elicited by PDT in various mouse tumor models, there is limited clinical evidence supporting this property. The number of appropriately designed clinical trials to evaluate the effectiveness of PDT in eliciting anti-tumor immunity remains very low. In our search across various databases, we have only found 13 clinical reports dealing to the immunostimulatory properties of PDT. The importance of the host immune system for the PDT efficacy in patients was first demonstrated in 2001. In this study, 32 patients with the pre-cancerous condition vulval intraepithelial neoplasia were treated with PDT using **5-ALA** (DL = 50–100 J/cm^2^). This study revealed a significant increase in CD8^+^ T cells at tumor biopsies among responders compared to non-responders, 3 months post-treatment^62^. In addition, reduced response was observed in HPV^+^ patients with HLA-1 loss/downregulation. Subsequent studies investigated the effects of **ALA-based PDT** on patients with basal cell carcinoma (BCC). In one of these studies, 17 patients who underwent topical **ALA-based PDT** (9 h ALA occlusion, 100 J/cm^2^) displayed enhanced ROS production by neutrophils isolated from peripheral blood 4 h after PDT (compared to neutrophils collected before PDT) thus indicating neutrophils activation. In contrast, peripheral blood lymphocytes showed a significant decrease in IL-1β and TGF-1 while IL-2, IL-6 and TNF-α concentrations remained unchanged^63^. A similar study from 2009 involving 15 patients with BCC and treated with **ALA-based PDT** (9 h ALA occlusion, 100 J/cm^2^) showed a prominent increase in immune infiltrates, with innate immune cells such as granulocytes (*e.g*. neutrophils) peaking at 4 h, while mast cells reached a maximum at 72 h post-PDT. In contrast, CD3^+^ T cells peaked at 24 h and CD68^+^ macrophages gradually increased up to 72 h, which was the last time point of analysis^64^. Another study published in 2009 involved BCC patients treated with either **ALA-based PDT** or PDT with **porfimer sodium**. For **ALA-based PDT, 5-ALA** was occluded for 4 to 24 h followed by illumination (100 to 260 J/cm^2^) 4 h after **5-ALA** application. For **porfimer sodium-based** **PDT**, **porfimer sodium** was administered intravenously (1 mg/kg), and light illumination (170 to 215 J/cm^2^) was performed 48 h after **porfimer sodium** infusion. This study demonstrated that both **5-ALA-PDT** and **porfimer sodium-PDT** enhance the recognition of the BCC tumor-associated antigen, Hedgehog-interacting protein 1 (Hip1), by peripheral blood leukocytes isolated 7–10 days after PDT. The immune response was found to be increased two-fold in 15 of the PDT-treated patients when compared to patients who underwent surgical lesion removal (4 patients)^65^. Another study with 12 BBC patients submitted to **5-ALA-based PDT** (3 h occlusion, 75 J/cm^2^) demonstrated enhanced number of epidermal Langerhans cells (skin antigen presenting cells) at tumor sites 1 week after PDT^66^.

Other PDT studies in the clinical setting have been conducted using the methyl derivative of **5-ALA, MAL**. In one of these studies from 2012, **MAL** cream (**Metvix®**) was applied to BCC patients (*n* = 8) followed by a LD of 37 J/cm^2^. Biopsies demonstrated rapid neutrophil infiltration observed as soon as 1 h post-PDT, which significantly increased at 24 h when compared with the baseline of untreated healthy skin. It was also observed that there was an increase on E-selectin, a cell adhesion molecule that is expressed on the surface of endothelial cells. The number of CD4^+^ and CD8^+^ T cells were also augmented after PDT but the differences were not statistically significant. This study also revealed that **MAL-PDT** significantly reduced the number of epidermal Langerhans cells at least until 24 h. The lack of T cells and DC infiltrates may be related to the short time points (24 h) at which these analyses were carried out^67^. In another study from 2017 using **MAL-PDT** (3 h **MAL** occlusion, 37 J/cm^2^) in ten patients of BCC, tumor biopsies revealed increasing levels of IFN-γ, IL-17, IL-23 and IL-22 at an early time point (0.5 to 2 h after PDT) compared to the baseline (before PDT), which suggest Th1 and Th17 immune responses. This was followed by decreasing levels at 1 week to 3 months after PDT^68^.

The effect of **porfimer sodium-based PDT** (PS = 1 mg/kg; DLI = 48 h; LD = 80 J/cm^2^) on regulatory CD4^+^ CD25^+^ CD127^−^ FoxP3^+^ T cells (Treg) was investigated in eight patients with invasive esophageal squamous cell carcinoma. The results published in 2014 showed that the number of Tregs in the blood collected 7 and 14 days after PDT increased, but their suppressive activity was significantly inhibited. Tumor biopsies revealed that Tregs were reduced at day 7 but returned to baseline levels 14 days after PDT. A slight but statistically significant increase in peripheral neutrophils granulocyte and monocytes was observed at day 7, but not of lymphocytes. This study also showed an accentuated increase in the pro-inflammatory IL6 (maximum at day 7) but not in IL-8, IL-10, and TGF-β cytokines^69^.

The number of Tregs was also evaluated after **temoporfin-based PDT**, but the specific PDT protocol was not reported. This study was published in 2017 and included nine patients with head and neck squamous cell carcinoma who had undergone multiple oncologic treatment regimens. The authors reported that PDT increased the number of CD4^+^CD25^+^CD39^+^ Treg and NK cells in the peripheral blood collected at 24 h, as well as 4 to 6 weeks, after PDT. Although not statistically significant, the number of CD4^+^ and CD8^+^ T cells decreased on blood while B cells slightly increased during the first 24 h after PDT. Additionally, serum concentrations of IL-6 and IL-10 were significantly elevated, peaking at 24 h, while HMGβ1 reached its maximum at 3 days. Perforin levels decreased, but other cytokines in analyses (IL-2, IL-4, IL-5, IL-6, IL-10, perforin, GM-CSF, IFN-γ, Granzyme A and B, MIP-1α, MIP-1β and TNF-α) remained unchanged^70^.

A recent study investigated the effects of PDT on 52 patients with advanced colon rectal cancer, who were divided into four groups: PDT group (*n* = 8), PDT + standard therapy (ST) group (*n* = 10), ST group (*n* = 19), and untreated group (*n* = 15). The PDT protocol involved the administration of **porfimer sodium** (5 mg/kg) with a DLI of 48 h and DL = 200 J/cm^2^. The overall survival of the PDT group or PDT + ST group was significantly longer compared to the other groups that did not received PDT. Before PDT, the number of immune cells in patients with stage III (*n* = 7) was normal or slightly low. However, after PDT, there was a significant decrease in total T cells, CD4^+^ T cells and CD8^+^ T cells, as well as in the expression of CD45RA (naïve T cells) and CD45RO (memory T cells) receptors on CD4^+^ and CD8^+^ T cells, both in peripheral blood and tumor tissues samples collected 48 h after PDT. Although there was no statistical difference, B cells and NK cells also decreased in most cases. Conversely, patients with stage IV had a low number of immune cells at baseline levels. However, 48 h after PDT, there was an increase in most of the immune cells analyzed. Immunohistochemical studies showed that many inflammatory cells and immune cells (CD3^+^ T cells, CD20^+^ B cells, CD4^+^ T cells, CD8^+^ T cells, and macrophages) significantly infiltrated into the tumor tissue after PDT in both stage III and IV CRC patients^71^.

The activation of anti-tumor immunity is of utmost significance, not only for its ability to regulate residual cancer cells that evade PDT treatment at the primary tumor site, but also for its potential to identify and eliminate metastases in non-irradiated areas. Although a significant body of preclinical studies substantiated this notion by showing the abscopal control of distant metastases^6,38,45,46,48^, clinical evidence in humans remain scarce. One of the first clinical report describing the regression on untreated distant tumors after PDT dates from 2007. A 64-year-old Chinese man with histologically proven multifocal angiosarcoma of the head and neck was submitted to four PDT sessions within 21 months using **chlorin e6 (Fotolon®)**. The first and second treatments were carried out at head and neck lesions using high PDT regimen (PS = 5.7 mg/kg, LD = 200 J/cm^2^, fluence rate = 100 to 150 mW/cm^2^). This led to strong necrosis within 48 h post-PDT. Ten months later, new lesions had appeared on both upper limbs which were treated with an intermediate PDT dose (PS = 4.0 mg/kg, LD = 100 J/cm^2^ and 200 J/cm^2^, fluence rate = 82 mW/cm^2^). This resulted in tumor eradication but notably, spontaneous remission of neighboring and untreated lesions was observed 2–4 months after PDT. A last PDT treatment was carried at the head and neck region owing to recurrent lesions (PS = 2.0 mg/kg, LD = 65 J/cm^2^, fluence rate = 80 mW/cm^2^) that results in tumor eradication, inflammation, and spontaneous remission of non-illuminated lesions. Immunobiological analysis revealed a shift from CD4^+^ T cells to CD8^+^ T cell infiltrate at 1 month after PDT^72,73^.

Two studies with patients with advanced breast cancer and treated with **porfimer sodium** have also reported regression of tumor lesions distant from the treatment field. In one of these studies involving 14 patients, tumor illumination (150 to 200 J/cm^2^) was performed 48 h after the iv administration of **porfimer sodium** (0.8 mg/kg). Remarkable, complete response of the illuminated tumors was attained in 9 of the 14 patients despite some wound complications. The authors also reported regression of several tumor lesions outside of the field of illumination, 4 to 6 weeks after PDT^74^. In the other study, **porfimer sodium** (0.8 mg/kg) was administered 48 h before tumor illumination. Two patients received 100 J/cm^2^ and seven received 50 J/cm^2^ delivered over 24 h. From these patients, six had complete or partial clinical response. TUNEL assay was performed in eight patients and in all of them, tumor apoptosis was observed. Of note, two patients had complete relapse of untreated tumor nodules^65,75^.

Overall, the observations made in patients are consistent with preclinical findings in a variety of mouse models. Typically, it is observed an initial inflammatory response that is characterized by an increase in IL-6 and HMGβ1 in blood. This response is accompanied by neutrophilia and neutrophil infiltration into the tumor within the first 48 h. The increasing levels of IFN-γ, IL-17, IL-23, and IL-22 are also consistent with preclinical observations showing Th1 and Th17 immune responses, rather than Th2. These initial responses decline after approximately 1 week, indicating a transient inflammatory state that then evolves into acquired immunity. Macrophages and mast cells appear at the tumor site after 48–72 h, while DCs are detected after 1 week. T cells are detected at tumor sites as soon as 24 h, but also after 1 month. Peripheral T cells have enhanced capability to recognize tumor antigens 7–14 days after PDT. CD8^+^ T cell infiltrates are correlated with better responders to PDT, while HLA-1 loss/downregulation is correlated with non-responders. Immunosuppressive cytokines IL-10 and TGF-β, appear to increase with PDT, which might indicate compensatory mechanisms to avoid an exacerbated immune reaction with deleterious effects^62–75^. The clinical studies mentioned above also support the preclinical notion that PDT regimens using lower light doses and/or light fluences might lead to stronger anti-tumor immune responses^46^. Finally, abscopal control of metastases outside of the field of illumination is also reported in a few patients which highlighted the benefits of local therapies with immunomodulatory properties.

This clinical data is still limited. Hence, there is a requirement for well-designed clinical studies to investigate the impact of PDT on the immune system, which will enable us to enhance our understanding on the mechanism of anti-tumor immunity triggered by PDT and eventually employ this knowledge to improve clinical outcomes.

## Clinical trials of PDT for cancer treatment initiated in the last decade

In this Trial Watch, a comprehensive summary of all clinical trials initiated in the past ten years is provided. Our research on ClinicalTrials.gov utilized the keywords “cancer” and “photodynamic therapy” and covers the period from March of 2013 to March of 2023. After excluding trials with a withdrawn status, our search yielded 174 results. From these, we further excluded studies that did not specify the photosensitizer or those involving non-cancer conditions (e.g., port-wine stains) as well as two trials related to extracorporeal photochemotherapy. Our selection criteria resulted in 132 studies, which we organized into five tables for easy reference. Table 2 summarizes clinical trials of PDT for cancer treatment with photosensitizers while Table 3 focuses on PDT trials using the pro-drugs, **ALA** and its derivatives, for cancer treatment. Table 4 provides an overview of the clinical trials investigating the use of PDT for cancer treatment including its effects on immune responses. Table 5 refers to clinical trials investigating the combination of PDT with immune checkpoint blockers (ICBs). Finally, we also included a table in the supplement material that summarizes clinical trials of PDT related to skin disorders that may progress to cancer if left untreated (Table S1).

**Table 2.** Clinical trials involving PDT for cancer that have started between March of 2013 and March of 2023.

Photosensitizer	DLI	Cancer	Phase	Status	Country	Observations	Reference	Study start
Porfimer sodium	24–48 h	Malignant mesothelioma; Non-small cell lung carcinoma with pleural disorder	I	Recruiting	USA	Intraoperative PDT; PS IV administ.	NCT03678350	September 2021
	48–50 h	Non-small cell lung cancer; Lung metastasis	Early I	Unknown	Taiwan	Combination with Fotolon® ethiodized oil to enhance light deliver; PS IV administ.	NCT04753918	March 2021
	2–4 h	Locally advanced lung carcinoma; Non-small cell lung carcinoma; Small cell lung carcinoma; Lung Cancer AJCC v8 (Stage III/IIIA/IIIB/IIIC)	I/II	Recruiting	USA	Ultrasound-guided transbronchial needle-delivered interstitial PDT; PS IV administ.	NCT03735095	February 2020
	N/A	Non-small cell lung cancer	N/A	Terminated	USA	Combination with Argon plasma Coagulation; PS IV administ.; Slow accrual	NCT03564054	October 2018
	48 h	Lung cancer and metastasis	I	Completed	USA	Prior to surgical resection; PS IV administ	NCT03344861	August 2017
	48 h	Non-small cell lung cancer; Lung Metastasis	I	Completed	USA, Canada	Interstitial PDT with electro navigational bronchoscopy; PS IV administ.	NCT02916745	January 2017
	24 h	Malignant pleural mesothelioma	II	Completed	France	Intra-pleural PDT; Combination with chemotherapy and surgery; PS IV administ.	NCT02662504	January 2016
Porfimer sodium	24–48 h and 48 - 72h	Esophageal adenocarcinoma (Stage I, II, III); Esophageal cell carcinoma (Stage I, II, III)	III	Unknown	China	Endoscopic PDT, PS IV administ.	NCT02628665	October 2015
	24 h	Recurrent high-grade gliomas	II	Terminated	USA	PS IV administ.; insufficient enrolment	NCT01966809	June 2015
	48 h	Hilar cholangiocarcinoma	III	Terminated	USA	A 2^nd^ illumination cycle was planned after 96–120 h if 1^st^ illumination did not cover the entire tumor; PS IV administ; low accrual	NCT02082522	November 2014
	24 h	Epithelioid malignant pleural mesothelioma	II	Recruiting	USA	Radical pleurectomy with intra-operative PDT and post-operative chemotherapy; PS IV administ.	NCT02153229	May 2014
	48 h	Head and neck	II	Terminated	USA	Image-guided interstitial PDT in combination with chemotherapy; PS IV administ.; study no longer has an active IDE	NCT02068157	April 2014
	48 h	Advanced rectal cancer	II/III	Suspended	China	PDT through colonoscopy *vs* chemotherapy; PS IV administ.; modifying the clinical trials	NCT01872104	August 2013
	48 h	Cholangiocarcinoma	N/A	Suspended	China	PDT through T-tube *vs* biliary drainage; PS IV administ.; modifying the clinical trials	NCT01859169	June 2013
Porfimer sodium	1, 3, 8, and 21 days	Acinar cell adenocarcinoma of the pancreas; Duct cell adenocarcinoma of the pancreas; Pancreatic Cancer (Stage III)	I	Completed	USA	Combination endoscopic ultrasonography-guided PDT with gemcitabine hydrochloride; PS IV administ.	NCT01770132	April 2013
	24 h	Recurrent pediatric brain tumor	I	Completed	USA	PS IV administ.	NCT01682746	March 2013
Hematoporphyrin	48–72 h	Cholangiocarcinoma	N/A	Not yet recruiting	China	Combination with sonodynamic therapy; PS IV administ.	NCT05580328	December 2022
	48 h	Esophageal carcinoma in Situ AJCC V7	N/A	Not yet recruiting	China	PDT vs Endoscopic submucosal dissection; PS IV administ.	NCT05208775	March 2022
	24 h	Cholangiocarcinoma non-resectable	N/A	Recruiting	China	PS IV administ.	NCT04860154	April 2021
Polyhematoporphyrin	N/A	Hilar cholangiocarcinoma	N/A	Completed	Austria	N/A	NCT02504957	July 2015
Verteporfin	N/A	Recurrent prostate cancer	I/II	Recruiting	USA, Canada, UK	Interstitial PDT using SpectraCure P18 System for illumination; PS IV administ.	NCT03067051	March 2017
	1 h	Advanced pancreatic carcinoma; Locally advanced pancreatic carcinoma; Metastatic pancreatic carcinoma; Pancreatic neoplasm; Pancreatic carcinoma; Pancreatic cancer AJCC v8 unresectable (Stage II, IIA, IIB, III, IV)	II	Recruiting	USA	Endoscopic-ultrasound guided PDT; PS IV administ.	NCT03033225	December 2016
	N/A	Metastatic breast cancer	II	Unknown	USA	Continuous low-irradiance PDT	NCT02939274	October 2016
Deuteporfin	6 h and 9 h	Cholangiocarcinoma	II	Terminated	China	Business decision	NCT02955771	May 2017
Temoporfin	72 h	Cholangiocarcinoma	II	Recruiting	China	PS IV administ.	NCT03003065	March 2014
	N/A	Recurrent non-small cell lung carcinoma (Stage IIA, IIB, IIIA, IIIB)	I	Completed	USA	PS IV administ.	NCT01854684	February 2014
Chlorin e6	3 h	Advanced hilar cholangiocarcinoma	II	Unknown	South Korea	PS IV administ.	NCT02725073	January 2016
HPPH	24h	Head and neck	II	Terminated	USA	Low accrual; PS IV administ.	NCT03090412	May 2018
	48 h	Esophageal cancer	I	Unknown	China	PS IV administ.	NCT03757754	June 2015
LUZ11	15 min	Head and neck cancer	I/II	Recruiting	Portugal	PS IV administ.	NCT02070432	February 2014
Padeliporfin	Immediate after IV infusion	Transitional cell cancer of renal pelvis and ureter	III	Recruiting	USA, Austria, France, Israel	Ureteroscope for optical fiber placement; PS IV administ.	NCT04620239	March 2021
	Immediate after IV infusion	Low risk prostate cancer	IV	Terminated	France	Interstitial PDT; PS IV administ.; low accrual	NCT03849365	January 2019
	Immediate after IV infusion	Upper tract urothelial carcinoma	I	Active, not recruiting	USA	Endoscopic-PDT; PS IV administ.	NCT03617003	August 2018
	Immediate after IV infusion	Intermediate risk prostate cancer	II	Active, not recruiting	USA	Interstitial PDT; PS IV administ.	NCT03315754	October 2017
	Immediate after IV infusion	Esophagogastric cancer with moderate to severe dysphagia	I	Completed	USA, Israel	Endoscopic-PDT; PS IV administ.	NCT03133650	April 2017
	Immediate after IV infusion	Renal cancer	I/II	Terminated	UK	PDT with CT imaging guidance; PS IV administ.; Concerns about post-VTP MRI results being conclusive	NCT01573156	May 2013
	Immediate after IV infusion	Localized prostate cancer	III	Completed	Mexico, Panama, Peru	Interstitial PDT, PS IV administ.	NCT01875393	March 2013
Photobac	24h	Glioblastoma multiforme of brain glioma, sarcomatous	I	Not yet recruiting	USA	Combination with surgery; PS IV administ.	NCT05363826	November 2022
Silicon phthalocyanine 4	N/A	Recurrent cutaneous T-cell non-Hodgkin lymphoma; Recurrent mycosis fungoides/sezary syndrome; Cutaneous T-cell non-Hodgkin lymphoma (Stage I/IIA); Mycosis fungoides/sezary syndrome (Stage IA/IIA/IB)	I	Completed	USA	PS topical administ.	NCT01800838	April 2013
TLD-1433	1 h *	Non-muscle invasive bladder cancer refractory to BCG	II	Recruiting	USA, Canada	Intravesical PDT; PS infusion into the bladder for 1 h	NCT03945162	August 2019
	1 h *	Non-muscle invasive bladder cancer refractory to BCG	I	Completed	Canada	Intravesical PDT; PS infusion into the bladder for 1 h	NCT03053635	December 2016
Hypericin	18–24 h*	Cutaneous T-cell lymphoma; Mycosis fungoides	II	Completed	USA	Topical PS administ.	NCT05380635	May 2022
	2–4 h	Peritoneal carcinomatosis	III	Unknown	Germany	Oral PS administ.	NCT02840331	July 2017

**Table 3.** Clinical trials involving PDT of cancers with 5-ALA and derivatives started between March of 2013 and March of 2023.

Photosensitizer	DLI	Cancer	Phase	Status	Country	Observations	Reference	Study start
5-ALA	2 h *	Non-muscle invasive bladder cancer	N/A	Not yet recruiting	China	Combination standard infusion chemotherapy and surgery; PS infusion to bladder for 2h	NCT05547516	September 2022
	N/A	Skin tumors and non-cancer skin disorders	N/A	Recruiting	China	PS topical administ. with piezoelectric drive microneedling	NCT05488860	July 2022
	6 h	Glioblastoma	II	Recruiting	Belgium, France	Intraoperative PDT; PS oral administ.	NCT04391062	September 2021
	3.5–4.5 h	Glioblastoma	II	Recruiting	Germany	Stereotactic PDT; PS oral administ.	NCT04469699	April 2021
	3h*	Superficial and nodular basal cell carcinoma	II	Recruiting	USA	PS topical administ. with jet injection	NCT04552990	September 2020
	N/A	Basal cell carcinoma; Basal cell nevus syndrome	I	Active, not recruiting	USA	Combination with oral Vit.D3 pre-treatment	NCT03467789	October 2018
	3 h*	Superficial basal cell carcinoma	III	Recruiting	USA	Illumination with BF-Foscan®; PS topical administ.	NCT03573401	September 2018
	3h*	Skin cancer non-melanoma; skin cancer sun damaged skin; Actinic keratoses	I/II	Recruiting	USA	PS topical administ.	NCT03110159	August 2017
	4 h	Glioblastoma	N/A	Completed	France	Per-operative PDT; PS oral administ.	NCT03048240	May 2017
	0 - 2h	Head and neck	I/II	Completed	India	PS oral administ. at 0, 1 and 2 h before illumination	NCT03638622	March 2017
	3 h*	Squamous cell carcinoma	N/A	Unknown	USA	Surgical excision *vs* PDT; PS topical administ.	NCT03025724	January 2017
	2.5 h*	Nonmelanoma skin cancers in organ transplant recipients	N/A	Completed	USA	PS topical administ.	NCT02751151	February 2016
5-ALA and MAL	N/A	Basal cell carcinoma	I	Completed	USA	ALA + Vismodegib *vs* MAL + Vismodegib	NCT02639117	November 2015
	3 h*	Basal cell carcinoma	III	Completed	Germany	BF-200 ALA (Ameluz ®) *vs* Methyl-aminolevulinate (Metvix ®); PS topical administ.	NCT02144077	January 2014
	4 h*	Superficial basal cell carcinoma	IV	Unknown	Netherlands	5-ALA *vs* MAL; PS topical administ.	NCT01491711	August 2013
5-ALA, MAL and HAL	N/A	Neoplasms; Basal cell carcinoma	I/II	Active, not recruiting	Finland	5-ALA *vs* MAL *vs* HAL; PS topical administ.	NCT02367547	March 2015
MAL	3h*	Superficial basal cell carcinoma; Bowen’s Disease	N/A	Completed	Belgium	Combination will full ablative CO2 laser *vs* fraction ablative CO2 laser; PS topical administ.	NCT03012009	September 2014

(*) Time of occlusion or infusion.

**Table 4.** Ongoing clinical trials to evaluate PDT-mediated anti-tumor immunity.

Photosensitizer	DLI	Cancer	Phase	Status	Observations	Country	Reference	Study start
Porfimer sodium	24-48h	Malignant mesothelioma, non-small cell lung cancer or any other malignancy with pleural disease	I	Recruiting	No information is provided regarding which immune markers are under analysis	USA	NCT03678350	September 2021
Porfimer sodiumInterstitial-PDT Interstitial-PDT	2-4h	Locally advanced lung cancer in the central airway Non-small cell lung carcinoma; Lung Cancer AJCC v8 (stage III, IIIA, IIIB, IIIC)	I/II	Recruiting	No information is provided regarding which immune markers are under analysis	USA	NCT03735095	February 2020
5-ALA	4h	Basal Cell Carcinoma	II	Recruiting	(*)	USA	NCT05020912	November 2021
5-ALAInterstitial-PDT Interstitial-PDT	3.5–4.5 h	Newly diagnosed supratentorial IDH wild-type glioblastoma.	II	Recruiting	PBMC, CD4+, CD8+ are analyzed in blood samples of each patient	Germany	NCT03897491	September 2021

(*) described above.

**Table 5.** Ongoing clinical trials to evaluate the combination of PDT with immune checkpoint blockers.

Photosensitizer	DLI	Cancer	Phase	Status	Country	Combination	Reference	Study start
Porfimer sodium	24–48 h	Non-small cell lung cancer	I	Recruiting	USA	Do not mention which immunotherapy	NCT04836429	March 2022
	48 h	Locally advanced or recurrent head and neck cancer	I/II	Recruiting	USA	Immunotherapy (nivolumab, or pembrolizumab) or chemotherapy (cisplatin or carboplatin and fluorouracil [5-FU]) and/or targeted agents (cetuximab) 7 days, 14 days, or 28 days after PDT	NCT03727061	July 2019
5-ALA	4–6 h	Malignant pleural mesothelioma	II	Recruiting	France	Nivolumab	NCT04400539	May 2022
Sinoporphyrin sodium	24 h	Esophageal squamous cell carcinoma	II	Not yet recruiting	China	Pembrolizumab	NCT05386056	December 2022
ASP-1929	24 h	Locoregional recurrent squamous cell carcinoma of the head and neck, with or without metastases	II	Recruiting	Taiwan	Pembrolizumab	NCT05265013	April 2022
	24h	Recurrent or metastatic squamous cell cancer of the head and neck; advanced or metastatic cutaneous squamous cell carcinoma	I/II	Active, not recruiting	USA	Pembrolizumab or Cemiplimab	NCT04305795	December 2020
RM-1995	24 h	Locally advanced cutaneous squamous cell carcinoma	Ia (monotherapy) Ib (Combination)	Recruiting	USA	Pembrolizumab	NCT05220748	March 2022

**Figure 2.**
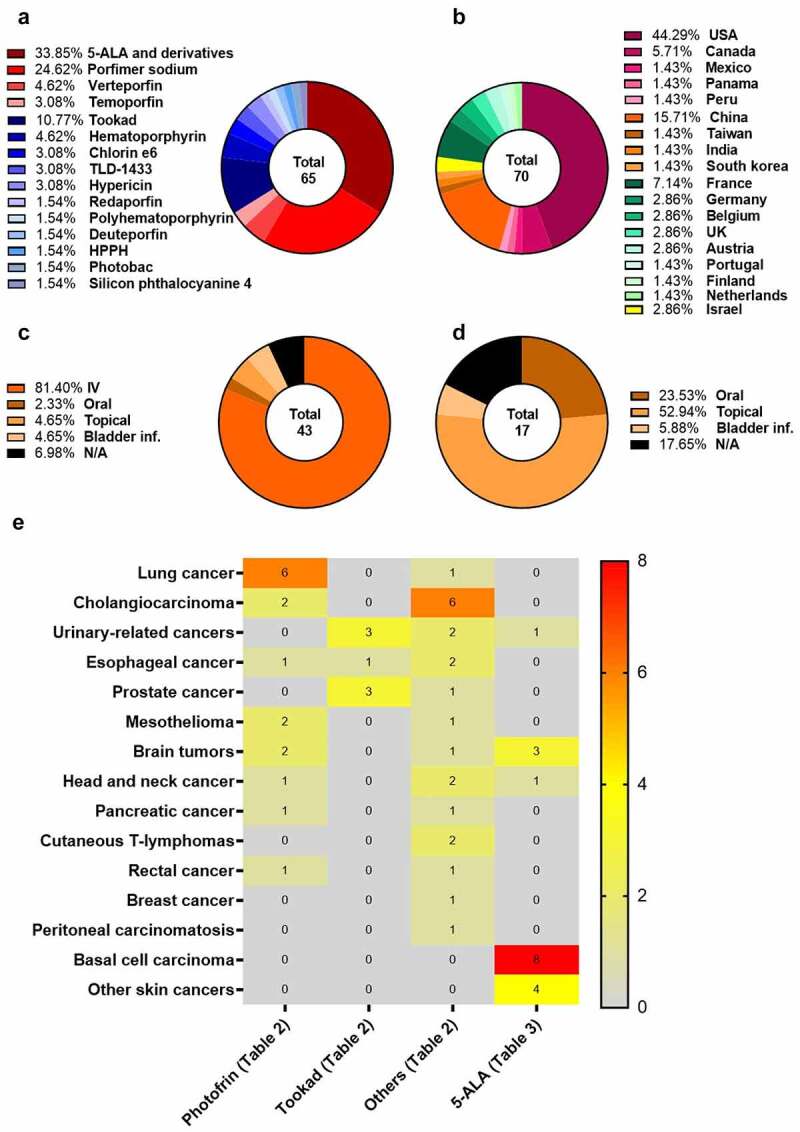
Comprehensive overview of clinical trials of PDT for cancer treatment. a) List and percentage of each photosensitizer used in clinical research, including **ALA** and its derivatives, based on Tables 1 and 2; b) Ranking of countries where the clinical trials are being conducted, based on Tables 1 and 2; c) Route of administration of photosensitizers in clinical trials, based on Table 2; d) Route of administration of **ALA** and its derivatives in clinical trials, based on Table 3; e) Heatmap showing the distribution of the photosensitizers, including **ALA** and its derivatives, in clinical trials across different types of cancer (Tables 2 and 3).

The most relevant information from Tables 2 and 3 is summarized in Figure 2, which provides an overview of the current state of clinical trials in PDT. It shows that **5-ALA** and its derivatives are the most commonly studied photosensitizing agents in clinical research, accounting for 34% of trials, followed by **porfimer sodium** at 25%. Interestingly, the majority of the clinical trials identified in the last decade have employed photosensitizers that have already been approved at least by one regulatory agency. The goal of these studies is to assess the effectiveness of PDT utilizing these photosensitizers for cancer types beyond those for which they are currently approved. Furthermore, these studies sought to explore the potential benefits of combining PDT with conventional treatments, such as chemotherapy or surgery. In addition, some of these clinical studies include the evaluation of technical parameters associated with the PDT protocol such as the use of fibers directly placed into the target tumors (interstitial PDT) and endoscopic procedures that can facilitate the placement of fibers toward the target tumors.

Only a relatively small proportion of clinical studies involve novel photosensitizers that have emerged from recent research. Examples of such molecules include, **deuteporfin**, a porphyrin derivative, the **ruthenium-based complex TLD1433** and the bacteriochlorins, **padeliporfin** and **redaporfin**. Bacteriochlorins, in particular, appear to represent a promising class of new chemical entities due to their strong near-infrared absorption that enables the treatment of deeper lesions. **Padeliporfin** (WST11, **Tookad®**) is a semi-synthetic molecule derived from bacteriochlorophyll α found in benthic bacteria. It exhibits high absorption at 763 nm (ε = 1.1 × 10^5^ M^−1^ cm^−1^) and fast body clearance, with a half-life in the range of a few minutes, which significantly limits the risk of skin photosensitivity^3^. Its fast clearance enables its use only in vascular-PDT protocols with tumor illumination occurring immediately after its infusion. Among the novel molecules without approval by any regulatory agency, **padeliporfin (Tookad®)** is the photosensitizer that has experienced more research, accounting for 11% of the trials (Figure 2a). It is worth noting that **Tookad®** was authorized by EMA in 2017 for the treatment of early-stage prostate cancer, but the FDA did not approve it^76–80^. In December 2021, Steba biotech withdrew the application submitted to EMA to extend the use of **Tookad®** in prostate cancer from the treatment of low-risk to intermediate-risk patients^81^. Currently, clinical trials also include patients with upper tract urothelial carcinoma, as listed in Tables 2 and 3.

**Redaporfin** (also known as LUZ11) is a synthetic fluorinated bacteriochlorin with high ROS quantum yield and high absorption at 749 nm (ε = 140 000 in ethanol)^82^. It is currently undergoing phase 1/2 clinical trials for advanced head and neck cancer^83^. Its pharmacokinetics profile is characterized by 1^st^ compartment (plasma) half-life of 0.5 h and 2^nd^ compartment half-life of 65 h which allows for applications in both vascular- and cellular-PDT protocols^84^. However, preclinical studies have demonstrated that vascular-PDT protocols yield the best therapeutic outcomes with cure rates close to 100% in mice bearing CT26 tumors or B16F10 tumors^6,85^. For this reason, clinical evaluation of **redaporfin** (0.75 mg/kg) involves tumor illumination (50 J/cm^2^) 15 min immediately after its intravenous infusion. Patients enrolled in **redaporfin** trials are advised to avoid sunlight for 3 days after administration of redaporfin. **Redaporfin** was granted Orphan Drug Designation from EMA for biliary tract cancer in 2016^86^.

The majority of the clinical trials are being conducted in USA (44%), followed by China (16%). European countries account for 24% of trials, with most of the trials taking place in France (Figure 2b). Based on the information collected, it can be observed that 81% of clinical trials involve the iv administration of the photosensitizers. However, there are exceptions such as **TDL-1433**, which involves bladder infusion, and **hypericin** that uses topical administration for cutaneous lymphomas or oral administration for peritoneal carcinomatosis, as well as **silicon phthalocyanine** that uses topical administration for cutaneous lymphomas (Figure 1c). Many of the clinical trials using the photosensitizers listed in Table 2 involve cellular-PDT protocols with DLI from 24 to 48 h (45%). Short DLIs of ≤15 min are only being used with **padeliporfin** and **redaporfin** (16%). DLIs between 1 and 9 h (8%) and of 72 h (8%) are also being used in clinical trials.

As expected, the vast majority of the clinical trials with **5-ALA** and its derivatives use topical administration as the main route (53%), which includes an occlusion varying from 2 to 4 h followed by tumor illumination. In addition, four trials involving oral administration of a drinkable formulation of **5-ALA** were found – three for glioblastoma and one for head and neck cancer. The drinkable form of **5-ALA (Gliolan®)** is an EMA orphan medicine since November 2002. It has been used for the visualization of malignant tissue during glioma surgery, enhancing tumor distinction from healthy brain tissue^87^. In these glioblastoma trials, **Gliolan®** is being tested in combination with surgery to remove remaining cancer cells, with administration taking place 2 to 6 h before surgery. **5-ALA** is also being tested for bladder cancer (with a 2-h infusion to the bladder) in combination with standard therapy. However, as expected, the primary target diseases for clinical research with **5-ALA** and its derivatives are skin-related disorders, such as basal cell carcinoma (eight clinical trials) and other non-melanoma skin cancers (four clinical trials). For photosensitizers strictly speaking, cholangiocarcinoma is the target of eight clinical trials: : two with **porfimer sodium**, two with **hematoporphyrin**, one with **polyhematoporphyrin**, one with **deuteporfin**, one with **temoporfin**, and one with **chlorin e6**. However, most of the clinical trials with **porfimer sodium** target lung cancer (six trials). Other cancers include mesothelioma, brain tumors, esophageal cancer, head and neck cancer, pancreatic cancer, and rectal cancer. As for **padeliporfin**, its clinical trials involve patients with cancer of the urinary system (three trials), prostate cancer (three trials), and esophageal cancer (one trial).

In addition, PDT with **5-ALA** and its derivatives are currently being investigated in 61 clinical trials for the treatment of pre-cancerous lesions, namely actinic keratoses (79%). Almost all of these studies utilize **5-ALA** (53%) or its methyl derivate, **MAL** (44%), which are already approved in the USA for the treatment of actinic keratoses (Figure S1). The majority of these studies are taking place in Europe (41%) followed by the USA (35%). The goals of these trials vary widely, ranging from testing different light wavelengths (blue, red, or daylight), to implement strategies to control the pain associated to the PDT-treatment (lidocaine, prilocaine, etc.), to enhance skin permeation (ablative fractional CO_2_ laser, microdermabrasion, etc.) and, PDT combination with other therapies (imiquimod, 5-fluorouracil, ingenol metubate, vitamin D, etc.) (Table S1).

## Ongoing clinical trials to evaluate PDT-mediated anti-tumor immunity

Clinical trials that include an analysis of the immune system after PDT are scarce. Since 2013, only four clinical trials have explicitly mentioned the analysis of immune cells with the goal of finding relationships between immune biomarkers and anti-cancer responses. In three of these trials, the main goal was the evaluation of the safety and/or anti-cancer effects, with the analysis of immune cells being a secondary goal (Table 4).

It is worth noting the clinical trial NCT05020912, which focuses on investigating the impact of PDT with **5-ALA** on the immune microenvironment of BCC. This trial plans to enroll 24 patients and is expected to be completed in September 2025. PDT treatment involves the topical occlusion of **5-ALA** during 4 h which is followed by light activation. In this study, each patient has one tumor that is treated with PDT, while another tumor remains untreated to serve as negative control.

Both treated and untreated tumors, as well as blood samples, are intended to be analyzed at: 1–3 days, 4–7 days, or 8–14 days post-PDT. This study aims to achieve the following goals:

- identify the altered expression of immune check point molecules and the time point at which these molecules reach their peak. For this, immunohistochemical studies are planned, using specific antibodies against PD-L1, PD-1, CTLA-4 as well as the newer TIGIT, TIM-3, and LAG-3;
- conduct a time course analysis of tumor-immune infiltrates: neutrophils (Gr1^+^ or MPO^+^); macrophages (F4/80^+^); MDSCs (CD33^+^); CD8^+^ T cells; Tregs (CD4^+^, FoxP3^+^, CD25^+^, CD127^−^) and NK cells (CD56^+^, CD16^+^) as well as to determine the ratio of CD8^+^ T cells to regulatory T cells;
- confirm the activation of systemic anti-tumor immune effects by analyzing CD8^+^ T cells in peripheral blood of the patients, collected before and after PDT treatment;
- evaluate the rate of **PpIX** accumulation in tumors as well as to determine the maximum levels of **PpIX** in tumors. This is done by noninvasive measurements of **PpIX** fluorescence using a dosimeter every 30 minutes during the 4 h occlusion;
- assess changes in the volume, color, and appearance of tumors at the Mohs surgery visit compared to the PDT visit;
- evaluate abscopal effects on tumors outside of the field of treatment;
- determine if PDT is associated with altered expression of immune- and cancer-associated RNA molecules using NanoString nCounter.

This study is likely the first clinical trial designed with the primary goal of systematically analyzing anti-tumor immunity after PDT. Its successful execution will increase our understanding regarding anti-tumor immune responses triggered by PDT at the clinical level. Clinical trials with other photosensitizers, and in cancers of different histological origins, will be important to gain a better understanding of the immune responses triggered by PDT in humans. In addition, this may help to solidify the notion of PDT as a form of immunotherapy.

## Ongoing clinical trials to evaluate the combination of PDT with immune checkpoint blockers

The highly immunosuppressive microenvironment of certain tumors is a significant barrier that greatly limits the success of anti-cancer therapies, including PDT. Despite strong evidence indicating that PDT can mediate good therapeutic effects, including anti-tumor immunity associated with abscopal effects, a significant number of patients do not respond to this treatment. Combining PDT with approaches that can boost the host immune system, namely immune checkpoint blockers, may improve therapeutic outcomes.

Overexpression of inhibitory checkpoint receptors is one of the mechanisms that tumors have developed to evade immune surveillance. The use of antibodies (known as immune checkpoints blockers, ICBs) to inhibit these cell surface receptors has revolutionized the treatment of cancer. Antibodies targeting the programmed-cell death-1 (PD-1 or CD279) and its ligand PD-L1, along with cytotoxic T-lymphocyte-associated protein 4 (CTLA4 or CD152) have been in clinical use for approximately a decade. The accumulated experience shows that ICBs are unprecedentedly successful but only in a small number of patients (10–40%). In some cases, they may even accelerate disease progression^88–90^. Different reasons account for such failure, such as the varied level of expression of inhibitory checkpoint receptors between different tumor types and among patients. In addition, different immune checkpoints can be simultaneously expressed on the same patients. Considering this, blockers of other immune checkpoints have been investigated. In 2021, an antibody targeting the co-inhibitory receptor lymphocyte-activation gene 3 (LAG-3 or CD223) received FDA approval. Other immune checkpoint blockers namely T cell immunoreceptor with Ig and ITIM domains (TIGIT) and T cell immunoglobulin and mucin-domain containing-3 (known as TIM-3) are also being investigated in clinical trials for different types of cancer.

The combination of PDT with ICBs is being explored in preclinical studies^59,91^. These studies included the administration of ICBs and of the photosensitizer independently but also the use of more sophisticated delivery platforms that enable the simultaneous delivery of both therapeutic agents^92^. The majority of these studies showed that combination schedules may increase the overall mouse survival compared to monotherapy, although instances of failure have also been reported^93^. Furthermore, abscopal effects have also been reported even with aggressive and highly immunosuppressive tumors, such as the 4T1 triple negative breast cancer tumor model^92,94^.

Although preclinical evidence supporting the benefits of combining PDT with ICBs is emerging, there is currently no reliable information on patients. To date, only three case reports have been published in which patients underwent multiple therapies such as, surgery, chemotherapy, targeted therapy, PDT and anti-PD-1 antibodies.

A case report was published from the clinical trial with **redaporfin** (NCT02070432), which described the case of a 62-year-old man with recurrent head and neck squamous cell cancer. The patient had failed to respond to radiotherapy, chemotherapy (carboplatin, paclitaxel), and cetuximab (an EGFR targeting antibody). From May to July 2016, he received three sessions of vascular **redaporfin-PDT** (0.75 mg/Kg, 50 J/cm^2;^ DLI = 15 min). The treatment resulted in a significant tumor regression however, four months later, tumor growth was detected at the border of the non-illuminated area, with a spot of malignant cells. This patient underwent partial surgical removal, followed by 33 cycles of Nivolumab (anti PD-1 antibody), which resulted in complete clinical response. As of today, the patient is still without signs of the disease^83^.

A 54-year-old male patient with esophageal cancer and distant metastasis, suffering from dysphagia after receiving two ineffective cycles of chemotherapy (doxetaxel, nedaplatin and cisplatin) was admitted to the hospital, where a metal stent was inserted into the esophagus. Three days later, the patients underwent four sessions of PDT (24 h apart) using **HpD derivatives** (5 mg/kg, 390 J; DLI = 24 h). The patient also received four cycles of chemotherapy (paclitaxel, cisplatin) and three doses of sintilimab (an anti-PD-1 antibody) and of anlotinib (VEGF-targeted antibody) every 3 weeks. The stent was removed after 7 months, and after 16 months, the patients showed no signs of tumor recurrence neither dysphagia^95^. Another case from the same Chinese hospital described a 72-year-old male patient with advanced gastric adenocarcinoma who was not responsive to surgery neither to chemotherapy. This patient received four PDT procedures using **HpD** in combination with chemotherapy, trastuzumab (HER2-targeted antibody) and pembrolizumab (an anti-PD-1 antibody). After a 7-month follow-up period, the patient showed no signs of recurrence or metastases^96^.

The multitude of therapies that these patients underwent makes it difficult to draw any conclusion about the benefits of combining PDT with ICBs. However, these cases do highlight the advantages of using multimodal combinations that incorporate multiple therapies in the treatment of advanced and recurrent cancer, even in elderly people. Therefore, clinical trials methodically designed to specifically evaluate the combination of PDT with ICBs are needed to determine the true benefit of such combinatorial approach. Our search in ClinicalTrial.org only identified seven relevant studies. Of these, two are currently underway at the Roswell Park Cancer Institute and are still recruiting patients. The study NCT03727061 enrolls patients with locally advanced or recurrent head and neck cancer (estimated *n* = 82). It aims to evaluate the safety and therapeutic effects of combining **porfimer sodium-based PDT** with standard therapy such as: chemotherapy (cisplatin or carboplatin and fluorouracil), targeted agents (cetuximab), or immunotherapy (nivolumab or pembrolizumab). Tumor illumination is performed 48 h after the administration of the photosensitizer by inserting fibers into the target tumors (interstitial PDT). Safety, objective response rate, progression free survival, overall survival, and changes in quality of life will be evaluated between patients receiving standard therapy alone and patient receiving standard therapy plus PDT. This study also aimed to investigate the relationship between the response rate and the levels of serum alkaline deoxyribonuclease (DNase) activity, a circulating biomarker, as well as immune markers. However, it is not specified which immune markers will be evaluated. The other study, NCT04836429, aims to evaluate if **porfimer sodium-based PDT** performed after Video-Assisted Thoracic Surgery (VATS) can be used to enhance the responses of subsequent treatment with immunotherapy targeting the PD1-PDL1 axis. **Porfimer sodium** is administered 24–48 h before of VATS, and tumor illumination is performed after tumor removal. This study targets patients (estimated *n* = 16) with non-small cell lung cancer with pleural disease that are under treatment with chemotherapy with no disease progression and with PDL1 expression < 50%. In addition to the objective response rate, progression-free survival and overall survival, this study also aims to evaluate changes in the immune phenotype of peripheral blood CD8^+^ T cells and in platelet-to-lymphocyte ratio.

Similar to the previous study, NCT04400539 also aimed to evaluate the safety and effectiveness of PDT with VATS and subsequent immunotherapy using Nivolumab, an anti-PD-1 antibody. This study admits patients with malignant pleural mesothelioma who have relapsed after treatment with platinum-based doublet of chemotherapy, including pemetrexed (estimated *n* = 20). The PDT protocol of the trial involves the oral administration of **5-ALA** (20 mg/kg) followed by VATS 4 to 6 h later. Afterward, six cycles of illumination (total LD = 25 J/cm^2^) of the pleural are performed. Each cycle lasts 2.5 min followed by intervals of 2 min to enable better tissue oxygenation. Nivolumab is administered 7 to 10 days after the VATS and PDT procedures are administered again every two weeks, for up to two years. Safety, objective response rate, progression-free survival, overall survival, changes in quality of life and chest pain are intended to be evaluated.

There are currently two ongoing clinical trials to evaluate the combination **AS-1929-based PDT** (cetuximab-targeting IR700) with antibodies targeting PD-1 (Pembrolizumab or Cemiplimab). NCT04305795 is being conducted in the USA and aims to evaluate the combination in patients with recurrent or metastatic squamous cell cancer of the head and neck or advanced or metastatic cutaneous squamous cell carcinoma with positive expression for PD-L1 (estimated *n* = 74). ICBs are administered every three weeks on days 1 and 22 of each 6-week cycle, while **ASP-1929** is administered intravenously on day 8 of each 6-week cycle. Tumor illumination is performed 24 h later. This treatment schedule can be maintained for up to two years. This same treatment schedule is also being evaluated in another trial, NCT05265013, that takes place in Taiwan and enrolls patients with locoregional recurrent squamous cell carcinoma of the head and neck, with or without metastases (estimated *n* = 33). This trial aims to measure several parameters related to pharmacokinetics and the presence of anti-drug antibodies. Both trials are evaluating safety, tolerability, objective response rate, progression-free survival, overall survival and duration of response.

NCT05220748 is a clinical trial also involving a bioconjugate of **IRDye® 700**, specifically the conjugate of **IRDye® 700** to an anti-CD25 antibody, known as **RM-1995**. This study involves patients with recurrent cutaneous squamous cell carcinoma or head and neck squamous cell carcinoma (estimated *n* = 36). This trial includes a first phase to assess the safety, tolerability, maximum tolerated dose and maximum administered dose, pharmacokinetics, pharmacodynamics and preliminary efficacy of **RM-1995** alone. The next phase of the study will assess the effectiveness of combining **RM-1995-based PDT** with pembrolizumab (anti-PD-1 antibody). In this combination protocol, patients receive an infusion of Pembrolizumab (200 mg) one week prior to PDT treatment. **RM-1995** is then administered via infusion followed by tumor illumination approximately 24 h later.

Lastly, NCT05386056 involves a new photosensitizer, **sinoporphyrin sodium**, which is a derivative of **porfimer sodium**, more precisely a porphyrin dimer connected by an ether bond. This study aims to evaluate the effects and safety of combining **sinoporphyrin sodium-based PDT** with pembrolizumab (anti-PD-1 antibody) in patients with metastatic esophageal squamous cell carcinoma that have failed at least one line of standard therapy (estimated *n* = 54). In this study, the photosensitizer is administered intravenously and the primary tumor is irradiated after 24 h. One administration of pembrolizumab is performed each 3 weeks up to 35 administrations. The trial aims to evaluate safety, objective response rate, progression-free survival, overall survival and changes in the quality of life.

## Concluding remarks

We have identified a significant number of clinical trials on PDT for cancer treatment that have been initiated in the last decade, demonstrating that the field is actively evolving. However, most of these trials utilize photosensitizers that are already in the market which are known to have some limitations. The aim of most of these clinical studies is often incremental, focusing on improving current protocols through the use of interstitial fibers and endoscopic techniques, combination with standard therapies, or application in cancers with limited therapeutic options.

Despite the therapeutic benefits of PDT, which have been acknowledged for over 50 years, with the exception of dermatological applications, PDT did not become a first-line therapy for any specific type of cancer. There are several reasons that may account for this limited acceptance. First, PDT is a drug combination product that involves a certain level of complexity due to its multidisciplinary nature. This makes the development process, from research to clinical translation and regulatory approval, more interdisciplinary and dismaying. Moreover, the success of PDT in treating skin and mucosal oncological disorders strengthens the bias toward the treatment of superficial lesions and niche-applications. For these reasons, major pharmaceutical companies are not finding attractive to invest in the development and commercialization of new PDT strategies^97^. This explains the modest pipeline of innovative photosensitizers in clinical evaluation. PDT involves technical details of different fields, such as pharmacology, the spectroscopy of photosensitizers, light wavelength, light source, drug-to light intervals and methods of light delivery, that make it challenging to conduct a standardized systematic review to compare different PDT studies or, even more challenging, to compare PDT with other therapies. Guidelines are needed to ensure that PDT studies are properly reported and can be compared. Secondly, PDT is often used as a last resort after other standard treatments have failed, which leads to high variability among patients and treatment designs involving multiple combinations approaches. Finally, the number of patients enrolled in PDT treatments is still low, which limits the amount of available data for review. Thereof, it is important to have more clinical data showing the benefits of PDT in cancer treatment. This will increase awareness of this therapeutic tool among healthcare professionals.

A question that deserves further reflection is which oncological targets could benefit the most from PDT. PDT is often indicated to treat advanced cancer patients that no longer respond to standard therapies. Given the increased performance of standard therapies and the progress of immunotherapies, the profile of these patients is evolving to patients with higher tumor burden and more compromised health when they become eligible for treatment with PDT. This places PDT in an increasingly difficult segment. The use of PDT at an early stage of the disease should increase its success rate and acceptance. This approach was followed by **Tookad-based PDT** that obtained approval for low-risk prostate cancer. However, in this case it is difficult not to elicit adverse effects that negatively impact patients’ quality of life at a time when active surveillance is acceptable. The withdrawal of **Tookad®** application to extend its use from low-risk to intermediate-risk patients shows that this path to reach a more attractive market is very risky. **Cetuximab saratolacan** followed the more classical approach to address advanced cancer and has recently been approved for the treatment of advanced stage head and neck cancer in Japan. However, it remains unclear how widely accepted and effective **cetuximab saratolacan** will be, highlighting the importance of implementing an active pharmacovigilance program to accurately evaluate its safety and effectiveness.

A wider adoption of PDT requires both a change in perception and a nuance in strategy. PDT must make a convincing case for the benefits of drug-device combinations. The combination should be regarded as the best of both worlds rather than a niche. PDT is uniquely placed to benefit from the increasing sophistication of devices, including robotics, connectivity and interface with artificial intelligence. This is not possible for drugs. However, only drugs have a size commensurable with targets and markers of disease. Drug-device combinations, namely PDT, have the intrinsic ability to reach the target precisely, extract information from the target and adapt the therapy to the target to obtain the best clinical results.

The acceptance of PDT as an immunogenic anti-tumor treatment modality by the scientific and medical communities could be a turning point for PDT^98^. Despite a large body of preclinical evidence showing the anti-cancer immunomodulatory properties of PDT, it is surprising how few of these findings have been translated into clinical applications. Randomized clinical trials with parallel group assignments and sufficient patient numbers to ensure statistical power are necessary. Clinical investigation of PDT, either alone or in combination with immunotherapy as is currently done with **redaporfin**, presents various layers of complexity, including practical, technological, and scientific issues^83^. For example, it is not yet clear how light doses and fluence rates impact the anti-tumor immunity mediated by PDT. While a few studies suggest that low PDT regimens may be more effective in triggering anti-tumor immunity, others have shown that anti-cancer immune responses can still be achieved with high fluence rates. When using combination protocols between PDT and immunotherapy, several practical questions need to be addressed. These include determining the appropriate sequential administration schedule, the optimal number of treatments and, which immune checkpoint blockade to use. Nevertheless, it is quite evident that PDT can have an immediate strong impact in a solid tumor, manifested by a significant reduction in tumor size and changes in the tumor microenvironment. Although specific details may have to be worked out for each tumor type, it seems that the use of PDT to treat or prime the primary tumor and stimulate immune responses, holds much promise to find synergies with immunotherapies that can manage the surviving cancer cells in a more immune-responsive organism. In order to establish the foundations of combinations between PDT and immunotherapies, it is crucial to incorporate measures of immune response in PDT clinical trials, namely examining changes in the number and activation state of immune cells, cytokines, and other relevant biochemical biomarkers (DAMPs) both at the tumor lesions (treated and untreated) and in the blood. Ideally, this should be carried out at different time points after PDT in order to cover the main immune effects, from innate to acquire immunity.

The immunomodulatory properties of PDT represent a major point of differentiation from standard therapies. This and the low response rate of patients to immunotherapies offer a historical opportunity to improve the management of cancer that PDT and immunotherapy communities should explore together for the benefit of cancer patients^99^.

## Supplementary Material

Supplemental Material
