# Supplementary material for: Trial watch: an update of clinical advances in photodynamic therapy and its immunoadjuvant properties for cancer treatment
Source: Oncoimmunology. 2023 Jun 18;12(1):2226535. doi: 10.1080/2162402X.2023.2226535 (PMC10281486; doi:10.1080/2162402X.2023.2226535)
Supplement: Supplemental Material [file KONI_A_2226535_SM3254.docx]

**Table S1.** Clinical trials involving photodynamic therapy of photodamaged skin started between March of 2013 and March of 2023.

| Photosensitizer | DLI | Cancer | Phase | Status | Country | Comb/Observa | Reference | Study start |
| --- | --- | --- | --- | --- | --- | --- | --- | --- |
| 5-ALA | N/A | Actinic keratoses | II | Not yet recruiting | USA | Combination with BLU -U Vs Chemical Peels | NCT04429308 | January 2023 |
|  | N/A | Photochemotherapy Actinic keratoses | Early I | Not yet recruiting | USA | Combination with Portable Apple Smart Phone Protoporphyrin IX Spectrometer Camera | NCT04223570 | December 2022 |
|  | 3h* | Actinic keratoses | III | Recruiting | USA | Combination with BF-200 ALA + red light LED lamp And Combination with vehicle + red light | NCT05662202 | December 2022 |
|  | 3h* | Actinic keratoses | I | Recruiting | USA | Red light LED lamp | NCT05060237 | December 2021 |
|  | 30 min and 3 h* | Actinic keratoses | N/A | Unknown | China | Painless PDT group applied for 30 min and conventional PDT group for 3h | NCT04396184 | May 2020 |
|  | 0.5-10h* | Actinic keratoses | I | Completed | USA | Red light LED lamp | NCT04319159 | March 2020 |
|  | 30 min* | Actinic keratoses | II | Completed | USA | Combination with Vit. D3 | NCT04140292 | January 2020 |
|  | 30 min* | Actinic keratoses | N/A | Completed | USA | Sunlight (2h) vs red light (10 min) | NCT03805737 | November 2019 |
|  | 3 and 4h* | Actinic keratoses Photodamaged skin | IV | Suspended | UK | Lack of appropriate personnel | NCT03963102 | October 2019 |
|  | 3h* | Actinic keratoses | IV | Recruiting | China |  | NCT03642535 | August 2018 |
|  | N/A | Actinic keratoses | N/A | Unknown | China | Combination with plum-blossom needling (PBN) | NCT03596619 | August 2018 |
|  | 4h* | Actinic keratoses | II | Completed | Germany | PD P 506 A (dermal patch loaded with 2 mg 5-ALA) | NCT03606122 | July 2018 |
|  | 15 min and 1h* | Actinic keratoses | I | Completed | USA | BLU-U PDT | NCT03322293 | December 2017 |
|  | N/A | Actinic keratoses | N/A | Active, not recruiting | USA |  | NCT03319251 | October 2017 |

*Table S1. (Continued)*

| Photosensitizer | DLI | Cancer | Phase | Status | Country | Comb/Observa | Reference | Study start |
| --- | --- | --- | --- | --- | --- | --- | --- | --- |
|  | 0 or 1h* | Actinic keratoses | N/A | Active, not recruiting | USA | BLU-U PDT | NCT03066843 | March 2017 |
|  | 25 min and 60 min* | Actinic keratoses | II | Completed | USA |  | NCT02632110 | March 2016 |
|  | N/A | HPV+ Cervical intraepithelial neoplasia and HPV+  Low-grade squamous intraepithelial lesions | II | Completed | China | PS topical administ. | NCT02631863 | March 2016 |
|  | 3 h* | Actinic keratoses | II | Completed | USA | BLU-U PDT | NCT02628236 | February 2016 |
|  | N/A | Basal cell nevus syndrome | N/A | Completed | USA | Blue light (Blu-U®) vs red light PDT (Aktilite™) | NCT02157623 | February 2016 |
|  | 3 h* | Lentigo maligna | IV | Completed | Finland | PS topical administ. | NCT02685592 | February 2016 |
|  | 3 h* | Actinic keratoses, pain | N/A | Completed | Sweden |  | NCT02644187 | December 2015 |
|  | 30 min and 1h* | Actinic keratoses | IV | Completed | USA | BLU-U PDT | NCT02622594 | October 2015 |
|  | 30 min* | Actinic keratoses | IV | Completed | Finland | Daylight PDT | NCT02464709 | June 2015 |
|  | N/A | Actinic keratoses | IV | Completed | USA | Daylight PDT | NCT03327831 | April 2015 |
|  | N/A | Actinic keratoses | N/A | Completed | USA | Combination with Antihistamine Cetirizine Hydrochloride | NCT02451579 | February 2015 |
|  | N/A | Cervical persistent high risk HPV infection;  Cervical intraepithelial neoplasia | II | Completed | China |  | NCT02304770 | January 2015 |
|  | 3 h* | Actinic keratoses | II | Completed | USA | BLU-U PDT | NCT02281136 | December 2014 |
|  | 1 h* | Actinic keratoses | N/A | Completed | USA | 10 min vs 20 min incubation | NCT02594644 | November 2014 |

*Table S1. (Continued)*

| Photosensitizer | DLI | Cancer | Phase | Status | Country | Comb/Observa | Reference | Study start |
| --- | --- | --- | --- | --- | --- | --- | --- | --- |
| 5-ALA | 1 h * | Actinic keratoses | II | Completed | USA | BLU-U PDT  Cryotherapy | NCT02239679 | September 2014 |
|  | N/A | Actinic keratoses |  | Completed | USA | BLU-U PDT | NCT02209012 | August 2014 |
|  | 3h* | Actinic keratoses | III | Completed | USA | BLU-U PDT | NCT02137785 | May 2014 |
|  | 3 - 5 h | Desmoids tumours | II | Recruiting | Israel | Combination with surgery; PS oral administ. | NCT01898416 | June 2013 |
| 5-ALA and MAL | N/A | Skin neoplasms | IV | Not yet recruiting | USA | AMELUZ® (10% ALA) vs LEVULAN® KERASTICK® (20%);PS topical administ. | NCT05359419 | May 2022 |
|  | N/A | Actinic keratoses | IV | Unknown | Spain |  | NCT02647151 | December 2015 |
| MAL | N/A | Actinic keratoses | N/A | Recruiting | Germany | Daylight PDT | NCT05725213 | November 2022 |
|  | 10 min* | Actinic keratoses | N/A | Recruiting | Belgium | Combination with Dermaris | NCT05522036 | January 2022 |
|  | N/A | Actinic keratoses | III | Completed | USA |  | NCT04269395 | April 2020 |
|  | N/A | Actinic cheilitis leukopatia erythropatia | II | Unknown | Brazil |  | NCT03990636 | October 2019 |
|  | 30 min* | Actinic keratoses | III | Completed | USA | MAL 16.8% and MAL Vehicle Cream; Daylight PDT (2h) | NCT04085367 | September 2019 |
|  | 3h* | Bowen’s disease | IV | Recruiting | Netherlands | PS topical administ. PDT vs 5-FU vs surgery | NCT03909646 | May 2019 |
|  | N/A | Actinic keratoses | IV | Completed | Germany | Daylight PDT | NCT03511326 | June 2017 |
|  | 3h* | Actinic keratoses | IV | Completed | South Korea | Combination with Lidocaine/prilocaine (5%) | NCT03731988 | February 2017 |
|  | 90 min* | Actinic keratoses | N/A | Completed | Brazil |  | NCT02878382 | October 2016 |
|  | 3h and 30 min* | Actinic Keratoses | N/A | Completed | France, Germany | MAL (3h)+Aktilite® Galderma vs MAL (30 min)+ PHOS ISTOS PD | NCT03076892 | September 2016 |
|  | N/A | Actinic keratoses | N/A | Unknown | Brazil | Daylight PDT | NCT03013647 | September 2016 |

*Table S1. (Continued)*

| Photosensitizer | DLI | Cancer | Phase | Status | Country | Comb/Observa | Reference | Study start |
| --- | --- | --- | --- | --- | --- | --- | --- | --- |
| MAL | 30 min* | Actinic keratoses  Sun damaged skin | II | Completed | Denmark | Ablative fractional CO2 laser vs microdermabrasion | NCT03006185 | August 2016 |
|  | 30 min* | Actinic keratoses  Photodamaged skin | IV | Completed | Germany | Daylight PDT | NCT02736760 | March 2016 |
|  | 2-3 h and 30 min* | Actinic Keratoses, Photodamaged skin | N/A | Active, not recruiting | Finland | Pulsed-dye laser+fractional-CO2-laser+Metvix® | NCT05456334 | March 2016 |
|  | 3 h* | Actinic dermatoses | I | Completed | South Korea | Combination with lidocaine/ prilocaine (5%) + MAL  150/350/500μm-AFL-PDT (ablative fractional laser-assisted photodynamic therapy) | NCT03325803 | September 2015 |
|  | N/A | Actinic keratoses | N/A | Completed | Australia, France, Italy, Netherlands, Spain, Switzerland, UK | Daylight-PDT | NCT02674048 | September 2015 |
|  | N/A | Actinic keratoses | III | Completed | France | BLU-U PDT | NCT02373371 | March 2015 |
|  | N/A | Actinic keratoses | II | Unknown | Canada | Combination with Ingenol metubate (Picato®) | NCT02354391 | January 2015 |
|  | N/A | Actinic keratoses | IV | Unknown | Netherlands | Combination with Imiquimod,5-fluoracilo, Ingenol metubate | NCT02281682 | November 2014 |
|  | 3 h and 30 min* | Actinic keratoses | N/A | Terminated | France | Decision of the investigator in the face of new scientific knowledge | NCT03076918 | September2014 |
|  | 2 h and 3 h | Actinic keratoses | I | Completed | South Korea | Combination with Lidocaine/prilocaine (5%)  2940- nm Er:YAG AFL pre-treatment | NCT02670655 | June 2014 |

*Table S1. (Continued)*

| Photosensitizer | DLI | Cancer | Phase | Status | Country | Comb/Observa | Reference | Study start |
| --- | --- | --- | --- | --- | --- | --- | --- | --- |
| MAL | 3 h* | Actinic dermatosis/keratoses | I | Completed | South Korea | Combination with Vit D | NCT02976727 | May 2014 |
|  | N/A | Actinic keratoses | N/A | Completed | China | Daylight-PDT and Maquet Power 500 LED surgery light | NCT02520700 | September 2013 |
|  | 30 min* | Actinic keratoses | N/A | Completed | Norway | Laser CO2 laser pre-PDT | NCT01898936 | August 2013 |
|  | N/A | Actinic keratoses | III | Completed | France, Germany, Netherlands | Natural daylight PDT vs Conventional PDT | NCT01821391 | July 2013 |
| MAL and HAL | N/A | Actinic keratoses | I/II | Completed | Finland | Daylight-PDT | NCT02149342 | May 2014 |
| Methylene blue | 15 min | Verruca Vulgaris | N/A | Recruiting | Egypt | Combination with IPL | NCT04620785 | January 2020 |

(*) Time of occlusion or infusion.


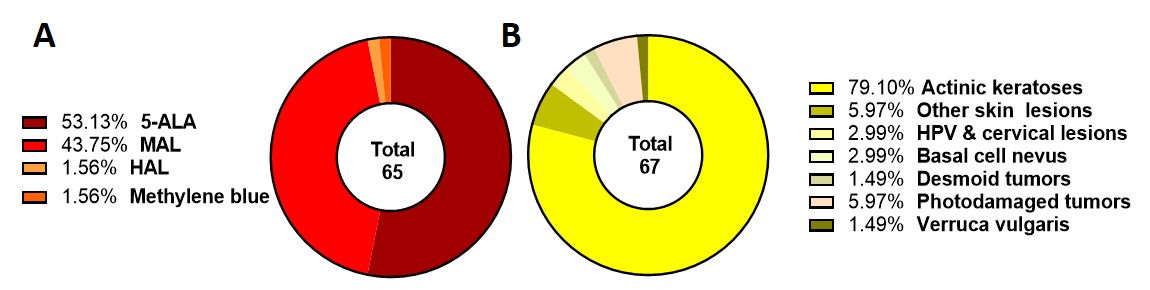


**Figure S1.** **Comprehensive overview of clinical trials of PDT for the treatment of pre-cancerous lesions.** **A)** List and percentage of each photosensitizing agent used in clinical research, based on Tables S1; **B)** types of pre-cancerous lesions under clinical investigation with PDT, based on Table S1.
